# Epigenetics and Autism Spectrum Disorder: Is There a Correlation?

**DOI:** 10.3389/fncel.2018.00078

**Published:** 2018-03-27

**Authors:** Adrien A. Eshraghi, George Liu, Sae-In Samantha Kay, Rebecca S. Eshraghi, Jeenu Mittal, Baharak Moshiree, Rahul Mittal

**Affiliations:** ^1^Department of Otolaryngology, University of Miami Miller School of Medicine, Miami, FL, United States; ^2^Dr. Kiran C. Patel College of Osteopathic Medicine, Nova Southeastern University, Fort Lauderdale, FL, United States; ^3^Division of Gastroenterology, Department of Medicine, University of Miami Miller School of Medicine, Miami, FL, United States

**Keywords:** epigenetics, autism spectrum disorder, DNA methylation, histone, transgenerational inheritance, microRNAs

Autism spectrum disorder (ASD) is the term for a range of development disorders caused by a combination of genetic and environmental factors (Kubota et al., 2012; Loke et al., 2015; Constantino and Marrus, 2017). ASD includes a “spectrum” of effects, skill detriments, and disability—including communication issues, limited interest in activities, and other aspects of work and life functionality. The current occurrence of ASD in the United States is about 1 in 68 children—an astonishing increase of over a factor of 100 compared to 2,000 (https://www.cdc.gov/ncbddd/autism/addm.html) (Zablotsky et al., 2015). Thus, it is imperative to develop novel treatment modalities for which understanding the pathogenetic factors underlying ASD is of utmost importance. Recently, the multigenic condition of ASD has been speculated to be dependent on epigenetic effects (Loke et al., 2015), although such exact factors remain unclear. Epigenetics refers to the heritable changes in gene expression without changing the underlying DNA sequence (Schiele and Domschke, 2017). In this opinion article, we will briefly discuss the recent advancements in understanding the contribution of epigenetic factors that can play a role in determining the predisposition to autism (Table 1). We emphasize that there is a need to include greater sample size and appropriate tissue type in order to better understand the role of epigenetics in ASD.

**Table 1:** A summary of epigenetic changes associated with Autism Spectrum Disorder (ASD).

**Epigenetic mechanism**	**Observation**	**Species**	**Tissue type**	**References**
DNA methylation	Lower methylation of Proline-rich transmembrane protein 1 (*PRRT1*) gene.	Human	Brain Tissue	Ladd-Acosta et al., 2014
DNA methylation	Higher methylation in placenta from ASD subjects by placental methylome analysis.	Human	Placenta	Schroeder et al., 2016
DNA methylation associated with maternal asthma	Hypermethylation of *FAM181A, CHFR*, and *AURKA* genes.	Human	Blood	Gunawardhana et al., 2014
DNA methylation associated with maternal asthma	Hypomethylation in *MAP8KIP3* and *NALP1L5*.	Human	Blood	Gunawardhana et al., 2014
Epigenetic proteins	Increased expression of Tet methylcytosine dioxygenases (*TETs*)-1,-2, and-3.	Human	Brain	Zhubi et al., 2017
Epigenetic proteins	Decreased expression of DNA methyltransferase 1 (*DNMT1*).	Human	Brain	Zhubi et al., 2017.
Transgenerational inheritance	Valproic acid (VPA) exposure leading to autistic-like phenotypes in male offspring.	Rodent	Live animals	Choi et al., 2016
Gene polymorphisms associated with variation in diet	Association of methylenetetrahydrofolate reductase (*MTHFR*) C677T polymorphism having ASD in children from countries without folic acid food fortification.	Human	Blood	Pu et al., 2013
Histone modifications	A common acetylome signature at >5,000 cis-regulatory elements observed in greater than 68% of syndromic and idiopathic ASD cases.	Human	Brain	Sun et al., 2016
microRNA (miRNA) dysregulation	Upregulation of hsa-miR-21-3p miRNA that targets neuronal genes downregulated in ASD.	Human	Brain	Wu et al., 2016
microRNA (miRNA) dysregulation	Downregulation of hsa_can_1002-m that regulates the epidermal growth factor receptor (EGFR) and fibroblast growth factor receptor (FGFR) signaling pathways involved in neural development and immune function.	Human	Brain	Wu et al., 2016

DNA methylation is one of the most well-known example of epigenetic regulation that generally correlates with close chromatin conformation and thus transcriptional silencing (Figure 1). DNA methylation has been implicated in the pathophysiology of neurological disorders including ASD (Ladd-Acosta et al., 2014; Ellis et al., 2017; Table 1). Though some recent studies reveal inconclusive findings, like how Retinoic acid-related orphan receptor-alpha (*ROR-*α*)* transcripts in blood lymphocytes have no observable differences between healthy and autistic children (Salehi et al., 2017), others demonstrate the emergence of replicated methylation biomarkers for ASD. Proline-rich transmembrane protein 1 (*PRRT1*) gene has exhibited lower methylation in the temporal cortex and cerebellum in autistic brains (Ladd-Acosta et al., 2014). On the other hand, human placental methylome analysis showed significantly higher methylation in ASD by pyrosequencing (Schroeder et al., 2016). However, there was a high level of variability between individuals in methylation status due to both sampling and individual variability. As placenta contains a heterogeneous mixture of various cell types, different ratios of these mixed populations of cell types between individual placental samples could be a source of the inter-individual variation observed in this study. Future studies employing cell sorting and data normalization approaches will help in confirming results of this study. A recent study showed that the mRNAs encoding the epigenetic proteins Tet methylcytosine dioxygenases (TETs)-1, -2, and -3 were increased, DNA methyltransferase 1 (DNMT1) was decreased, while methyl CpG binding protein-2 (MECP2) was unchanged in the frontal cortex in the brains of ASD subjects (Zhubi et al., 2017). Both MECP2 and DNMT1 protein binding to the RELN and GAD1 promoters was increased, possibly accounting for the decreased expression of both genes in ASD (Zhubi et al., 2017). From the results of these studies, it appears that epigenetic differences can potentially play a significant role in the development of ASD. Further research should pinpoint how the environmental factors contribute to alteration in gene expression that leads to predisposition to ASD. The epigenetic studies can be formulated through finding indicators of causative genetic as well as environmental factors, bringing forth greater insight into the physiological mechanisms that predispose children to ASD. The newly discovered biomarkers of ASD severity and prognosis will aid medical practitioners to more precisely fine-tune the plans of treatment.

**Figure 1:**
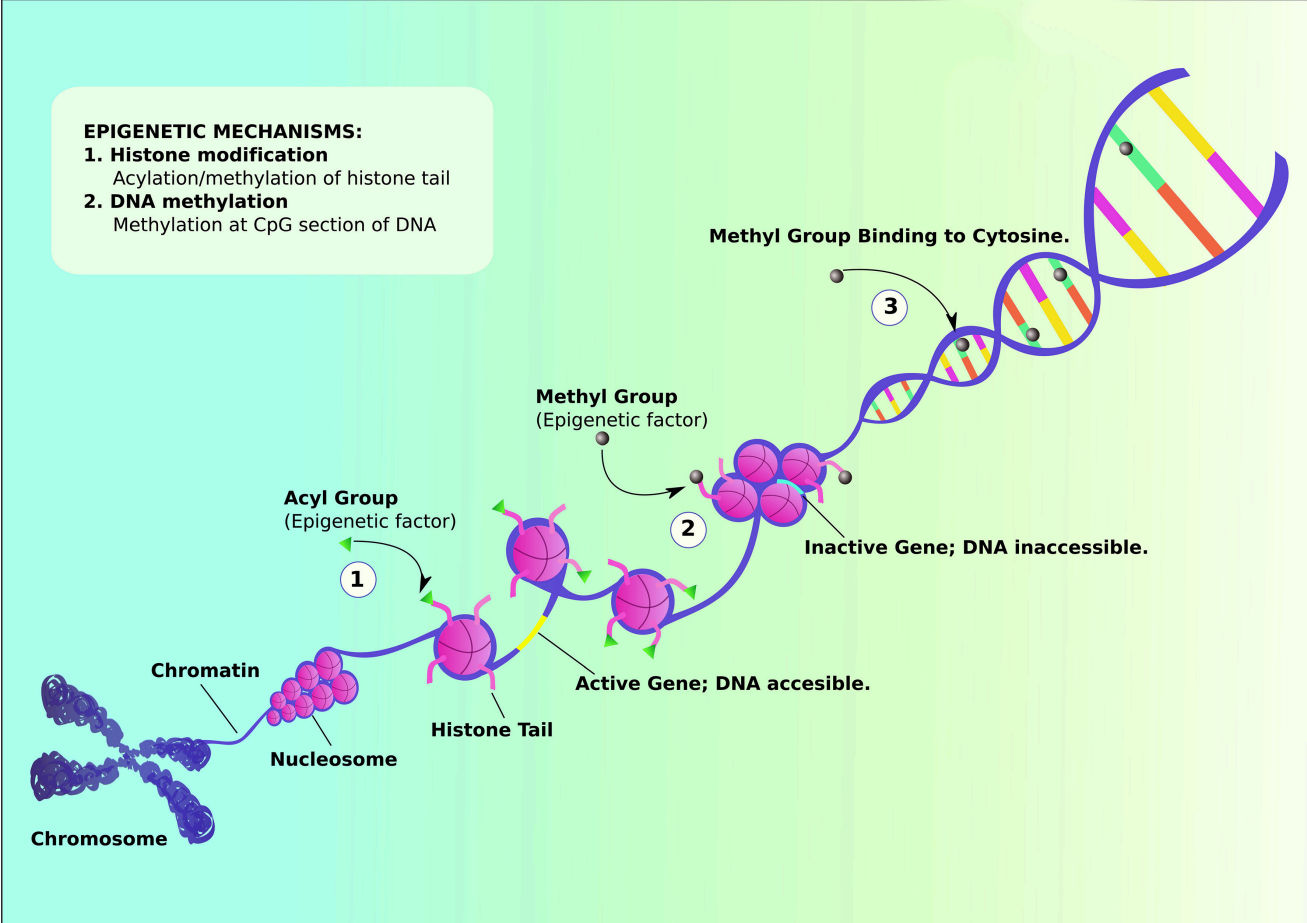
Schematic representation of Epigenetic mechanisms that can influence the genetic etiology. Histone modification can occur via either acylation or methylation of histone tail. (1) Acylation of histone tail leads to uncoiling of nucleosome, activating the gene and revealing DNA content to be modified. (2) On the other hand, methylation of histone tail coils nucleosomes tightly together, inactivating the gene and making DNA inaccessible for further modifications. (3) Methylation can also occur via binding methyl group to cytosine, suppressing transcription of certain genes. (Adapted from https://commonfund.nih.gov/epigenomics/figure).

In the past few years, many studies have focused their attention on transgenerational inheritance of ASD symptoms to propose epigenetic marks as a factor in ASD etiology. Valproic acid exposure in rodents has been shown to lead to autistic-like phenotypes in male offspring that can be epigenetically transmitted at least to the third generation (Choi et al., 2016). The autism-like behavioral phenotypes found in F1 persisted to the F2 and F3 generations. This study demonstrates that epigenetic inheritance could potentially play a role in the heritable nature of ASD. Environmental factors have also been shown to exert some control in epigenetic determination.

Variation in diet potentially contributes to epigenetic factor of ASD development. Polymorphism in methylenetetrahydrofolate reductase (MTHFR), coded by gene *MTHFR*, is associated with a significant increase in risk for developing ASD (Pu et al., 2013). The enzyme is essential for generating 5-methyl-tetrahydrofolatein, which plays a major role in DNA methylation during neural development. Among *MTHFR* polymorphisms, the C677T polymorphism was found to be specifically linked to increased risk of ASD in all comparison groups. A meta-analysis involving comparison of ASD prevalence in children from countries with folate acid fortified diet vs. those who are without food fortification showed a significant association of *MTHFR* C677T polymorphism in children from the latter group (Pu et al., 2013). In addition, through *in vitro* fertilization and embryo transfer in mice fed with protein-restricted and folic acid-supplemented diets, there appears to exist a significant degree of correlation between diet and ASD-like symptoms in offspring and maternal-protein restriction (Furuse et al., 2017). Indeed, epigenetic mechanisms involving DNA methylation, post-translational modifications of histone proteins, and transcriptional regulations (Figure 1) that are key to neurodevelopmental processes in-utero can possibly be affected by maternal lifestyle factors such as smoking, alcohol, obesity, and malnutrition (Barua and Junaid, 2015; Banik et al., 2017; Modabbernia et al., 2017).

Further, it has been demonstrated that epigenetics can influence activation of immune responses that can also determine susceptibility to ASD. Association between increased risk of ASD and maternal immune activation (MIA) during pregnancy has been suggested. A large self-report population study in Denmark found twofold increased prevalence of infantile autism in offspring of mothers that suffered influenza infection during pregnancy, and a threefold increased prevalence of ASD in children of mothers who experienced prolonged period of fever (Atladóttir et al., 2012). Aside from viral or bacterial infections, chronic asthma is one of the most common causes of maternal immune activation. Interestingly, epigenetic changes such as hypermethylation of *FAM181A, CHFR*, and *AURKA* genes, and hypomethylation in *MAP8KIP3* and *NALP1L5* immune genes in fetal blood samples were found in subjects exposed to maternal asthma (Gunawardhana et al., 2014). Furthermore, in a study that used a mouse model of maternal allergic asthma (MAA) to investigate alteration in microglia gene expression found significant amount of differentially expressed genes in MAA offspring which overlap the altered genes in human ASD cortex (Vogel Ciernia et al., 2017). These studies suggest the potential of epigenetic alterations by maternal immune responses that contribute to ASD development.

A study conducted a histone acetylome-wide association study (HAWAS) employing 257 postmortem samples from ASD and matched control brains that were subjected to H3K27ac chromatin immunoprecipitation sequencing (ChIP-seq) (Sun et al., 2016). A common acetylome signature at >5,000 cis-regulatory elements were observed in greater than 68% of syndromic and idiopathic ASD cases in prefrontal and temporal cortex although there was etiological heterogeneity. There was strong enrichment for genes related to ion channels, synaptic function, and epilepsy/neuronal excitability, all of which have previously been shown to be dysregulated during ASD. On the other hand, loci with decreased acetylation in ASD converged on pathways, such as digestive tract morphogenesis, chemokine signaling, HDAC activity, and immune processes related to microglia. As histone modifications are generally presumed to be stable in postmortem samples, this study laid a strong foundation to comprehend the role of epigenetics in ASD in the future investigations.

MicroRNAs (miRNAs) that are small non-coding regulatory RNAs which mediate mRNA destabilization and/or translational repression (Hammond, 2015; Mohr and Mott, 2015) has also been implicated in pathophysiology of ASD. miRNAs act as important regulators of gene expression as part of the epigenetic machinery (Soltanzadeh-Yamchi et al., 2018). A study performed genome-wide miRNA expression profiling in post-mortem brains from individuals with ASD and controls to determine the role of miRNAs in ASD (Wu et al., 2016). A shared pattern of miRNA dysregulation was observed in brains from ASD patients. Specifically, it include hsa-miR-21-3p, a miRNA of unknown CNS function that is upregulated in ASD and that targets neuronal genes downregulated in ASD. Another miRNA identified was hsa_can_1002-m, a previously unknown, primate-specific miRNA that is downregulated in ASD and that regulates the epidermal growth factor receptor (EGFR) and fibroblast growth factor receptor (FGFR) signaling pathways involved in neural development and immune function. Other studies have also implicated the role of miRNAs namely hsa-miR-29b, hsa-miR-219-5p, miR-146a, miR-221, miR-654-5p, and miR-656 in the pathophysiology of ASD (Sarachana et al., 2010; Nguyen et al., 2016). However, these studies have utilized olfactory mucosal stem cells (OMSCs) biopsied from ASD patients and control subjects or lymphoblastoid cell lines that necessitates the need to confirm the role of these miRNAs using brain samples.

Although the above-mentioned studies demonstrate the role of epigenetics in predisposition to ASD, some investigations does not support a role for epigenetics in autism. A study reported no difference in the proportion of global methylation between control and autistic brain (Ginsberg et al., 2012). This study do not find any change in the methylation of candidate genes. Therefore, it was concluded that changes in gene expression in autism may result from regulatory mechanisms different from gene methylation. These contradictory studies strongly make a case that there is a need to understand the role of epigenetics in autism.

There are a few considerations that should be taken into account while evaluating the role of epigenetics in ASD. Efforts to study genome-wide DNA methylation in relation to ASD should likely include a greater sample size and samples from same species in order to easily compare data from different studies. There is also a need to keep vigilant on standardizing the variability of phenotypes in test subjects to ensure a reduction in epigenetic complexities. With a larger sample size, one can better comprehend gene-gene and gene-environment interactions. The heterogeneity of ASD warrants genetic testing in large cohort of human patients that will help in establishing strong genotype-phenotype correlation as well as the role of epigenetics in ASD. With a collective systems-approach for future studies where findings are integrated and conglomerated, researchers can contemplate more accurate statistical tools and models for risk, diagnoses, and prognoses calculations. There is also a need to study the appropriate tissue when looking at epigenetic changes. The use of blood and buccal epithelial cells raises concerns about their similarity with the epigenome of neurological system. Also, there is a need to use either cerebrospinal fluid (CSF) or brain samples from a larger sample size to determine the role of epigenetics in ASD and confirm the validity of results. Nevertheless, the well-controlled studies can provide critical insights into the role of epigenetics in the pathophysiology of ASD. With the emergence of new state-of-the-art cutting edge technologies such as whole genome sequencing (WGS), it is possible to comprehend the complex role of epigenetics in ASD. The new genomic techniques allow more coverage of the genome, can provide better knowledge about ASD specific histone markers as well as can help in determining the contribution of non-coding regions such as intergenic regions and noncoding RNAs.

Autism researchers and individuals with ASD themselves have plenty to benefit from in learning through the fundamentals of epigenetics, as there exists a sense of robustness in the search for new epigenetic biomarkers that can be used for future interventions. Efforts in research of the epigenetics of autism are still uncertain and at times unrewarded, yet the prospective payoff of alleviating ASD-symptoms is too great a reward to not delve even further. A better knowledge about the role of epigenetics in autism will help in developing novel diagnostic biomarkers and treatment modalities leading to improved quality of life of many ASD patients and their families.

## Author contributions

All authors listed have made a substantial, direct and intellectual contribution to the work, and approved it for publication.

### Conflict of interest statement

The authors declare that the research was conducted in the absence of any commercial or financial relationships that could be construed as a potential conflict of interest.

## References

[B1] Atladóttir H. Ó. Henriksen T. B. Schendel D. E. Parner E. T. (2012). Autism after infection, febrile episodes, and antibiotic use during pregnancy: an exploratory study. Pediatrics 130, e1447–e1454. 10.1542/peds.2012-1107 23147969 PMC4451062

[B2] Banik A. Kandilya D. Ramya S. Stünkel W. Chong Y. S. Dheen S. T. (2017). Maternal factors that induce epigenetic changes contribute to neurological disorders in offspring. Genes 8:150. 10.3390/genes8060150 28538662 PMC5485514

[B3] Barua S. Junaid M. A. (2015). Lifestyle, pregnancy and epigenetic effects. Epigenomics 7, 85–102. 10.2217/epi.14.71 25687469

[B4] Choi C. S. Gonzales E. L. Kim K. C. Yang S. M. Kim J. W. Mabunga D. F.. (2016). The transgenerational inheritance of autism-like phenotypes in mice exposed to valproic acid during pregnancy. Sci. Rep. 6:36250. 10.1038/srep36250 27819277 PMC5098241

[B5] Constantino J. N. Marrus N. (2017). The early origins of autism. Child Adolesc. Psychiatr. Clin. N. Am. 26, 555–570. 10.1016/j.chc.2017.02.008 28577609

[B6] Ellis S. E. Gupta S. Moes A. West A. B. Arking D. E. (2017). Exaggerated CpH methylation in the autism-affected brain. Mol. Autism 8:6. 10.1186/s13229-017-0119-y 28316770 PMC5351204

[B7] Furuse T. Miyake K. Kohda T. Kaneda H. Hirasawa T. Yamada I. (2017). Protein-restricted diet during pregnancy after insemination alters behavioral phenotypes of the progeny. Genes Nutr. 12:1 10.1186/s12263-016-0550-2 28127411 PMC5248510

[B8] Ginsberg M. R. Rubin R. A. Falcone T. Ting A. H. Natowicz M. R. (2012). Brain transcriptional and epigenetic associations with autism. PLoS ONE 7:e44736. 10.1371/journal.pone.0044736 22984548 PMC3440365

[B9] Gunawardhana L. P. Baines K. J. Mattes J. Murphy V. E. Simpson J. L. Gibson P. G. (2014). Differential DNA methylation profiles of infants exposed to maternal asthma during pregnancy. Pediatr. Pulmonol. 49, 852–862. 10.1002/ppul.22930 24166889

[B10] Hammond S. M. (2015). An overview of microRNAs. Adv. Drug Deliv. Rev. 87, 3–14. 10.1016/j.addr.2015.05.001 25979468 PMC4504744

[B11] Kubota T. Miyake K. Hirasawa T. (2012). Epigenetic understanding of gene-environment interactions in psychiatric disorders: a new concept of clinical genetics. Clin. Epigenetics. 4:1. 10.1186/1868-7083-4-1 22414323 PMC3305338

[B12] Ladd-Acosta C. Hansen K. D. Briem E. Fallin M. D. Kaufmann W. E. Feinberg A. P. (2014). Common DNA methylation alterations in multiple brain regions in autism. Mol. Psychiatry 19, 862–871. 10.1038/mp.2013.114 23999529 PMC4184909

[B13] Loke Y. J. Hannan A. J. Craig J. M. (2015). The role of epigenetic change in autism spectrum disorders. Front. Neurol. 6:107. 10.3389/fneur.2015.00107 26074864 PMC4443738

[B14] Modabbernia A. Velthorst E. Reichenberg A. (2017). Environmental risk factors for autism: an evidence-based review of systematic reviews and meta-analyses. Mol. Autism 8:13. 10.1186/s13229-017-0121-4 28331572 PMC5356236

[B15] Mohr A. M. Mott J. L. (2015). Overview of microRNA biology. Semin. Liver Dis. 35, 3–11. 10.1055/s-0034-1397344 25632930 PMC4797991

[B16] Nguyen L. S. Lepleux M. Makhlouf M. Martin C. Fregeac J. Siquier-Pernet K.. (2016). Profiling olfactory stem cells from living patients identifies miRNAs relevant for autism pathophysiology. Mol. Autism 7:1. 10.1186/s13229-015-0064-6 26753090 PMC4705753

[B17] Pu D. Shen Y. Wu J. (2013). Association between MTHFR gene polymorphisms and the risk of autism spectrum disorders: a meta-analysis. Autism Res. 6, 384–392. 10.1002/aur.1300 23653228

[B18] Salehi M. Kamali E. Karahmadi M. Mousavi S. M. (2017). *RORA* and autism in the Isfahan population: Is there an epigenetic relationship. Cell. J. 18, 540–546. 10.22074/cellj.2016.4720 28042538 PMC5086332

[B19] Sarachana T. Zhou R. Chen G. Manji H. K. Hu V. W. (2010). Investigation of post-transcriptional gene regulatory networks associated with autism spectrum disorders by microRNA expression profiling of lymphoblastoid cell lines. Genome Med. 2:23. 10.1186/gm144 20374639 PMC2873801

[B20] Schiele M. A. Domschke K. (2017). Epigenetics at the crossroads between genes, environment and resilience in anxiety disorders. Genes Brain Behav. [Epub ahead of print]. 10.1111/gbb.12423 28873274

[B21] Schroeder D. I. Schmidt R. J. Crary-Dooley F. K. Walker C. K. Ozonoff S. Tancredi D. J.. (2016). Placental methylome analysis from a prospective autism study. Mol. Autism 7:51. 10.1186/s13229-016-0114-8 28018572 PMC5159983

[B22] Soltanzadeh-Yamchi M. Shahbazi M. Aslani S. Mohammadnia-Afrouzi M. (2018). MicroRNA signature of regulatory T cells in health and autoimmunity. Biomed. Pharmacother. 100, 316–323. 10.1016/j.biopha.2018.02.030 29453041

[B23] Sun W. Poschmann J. Cruz-Herrera Del Rosario R. Parikshak N. N. Hajan H. S. Kumar V.. (2016). Histone acetylome-wide association study of autism spectrum disorder. Cell 167, 1385–1397. 10.1016/j.cell.2016.10.031 27863250

[B24] Vogel Ciernia A. Careaga M. LaSalle J. M. Ashwood P. (2017). Microglia from offspring of dams with allergic asthma exhibit epigenomic alterations in genes dysregulated in autism. Glia 66, 505–521. 10.1002/glia.23261 29134693 PMC5767155

[B25] Wu Y. E. Parikshak N. N. Belgard T. G. Geschwind D. H. (2016). Genome-wide, integrative analysis implicates microRNA dysregulation in autism spectrum disorder. Nat. Neurosci. 19, 1463–1476. 10.1038/nn.4373 27571009 PMC5841760

[B26] Zablotsky B. Black L. I. Maenner M. J. Schieve L. A. Blumberg S. J. (2015). Estimated Prevalence of Autism and Other Developmental Disabilities Following Questionnaire Changes in the 2014 National Health Interview Survey. National Health Statistics Reports; no 87, National Center for Health Statistics, Hyattsville, MD. 26632847

[B27] Zhubi A. Chen Y. Guidotti A. Grayson D. R. (2017). Epigenetic regulation of RELN and GAD1 in the frontal cortex (FC) of autism spectrum disorder (ASD) subjects. Int. J. Dev. Neurosci. 62, 63–71. 10.1016/j.ijdevneu.2017.02.003 28229923 PMC5575980

